# HCV and Oxidative Stress in the Liver

**DOI:** 10.3390/v5020439

**Published:** 2013-01-28

**Authors:** Alexander V. Ivanov, Birke Bartosch, Olga A. Smirnova, Maria G. Isaguliants, Sergey N. Kochetkov

**Affiliations:** 1 Engelhardt Institute of Molecular Biology, Russian Academy of Sciences, Vavilov str., 32, Moscow 119991, Russia; E-Mails: aivanov@yandex.ru (A.I.); o.smirnova.imb@gmail.com (O.S.); kochet@eimb.ru (S.K.); 2 CRCL, INSERM U1052, CNRS 5286, Université de Lyon, 151, Cours A Thomas 69424 Lyon Cedex France; E-Mail: birke.bartosch@inserm.fr; 3 Department of Molecular Biology, Tumor and Cell Biology, Karolinska Institutet, Nobels väg 16 17177 Stockholm, Sweden; E-Mail: maria.issagouliantis@ki.se; 4 D.I. Ivanovsky Institute of Virology, Gamaleya Str. 16, 123098 Moscow, Russia; E-Mail: maria.issagouliantis@ki.se

**Keywords:** hepatitis C, oxidative stress, Nrf2/ARE pathway, antioxidant defense, iron homeostasis, regulation.

## Abstract

Hepatitis C virus (HCV) is the etiological agent accounting for chronic liver disease in approximately 2–3% of the population worldwide. HCV infection often leads to liver fibrosis and cirrhosis, various metabolic alterations including steatosis, insulin and interferon resistance or iron overload, and development of hepatocellular carcinoma or non-Hodgkin lymphoma. Multiple molecular mechanisms that trigger the emergence and development of each of these pathogenic processes have been identified so far. One of these involves marked induction of a reactive oxygen species (ROS) in infected cells leading to oxidative stress. To date, markers of oxidative stress were observed both in chronic hepatitis C patients and in various *in vitro* systems, including replicons or stable cell lines expressing viral proteins. The search for ROS sources in HCV-infected cells revealed several mechanisms of ROS production and thus a number of cellular proteins have become targets for future studies. Furthermore, during last several years it has been shown that HCV modifies antioxidant defense mechanisms. The aim of this review is to summarize the present state of art in the field and to try to predict directions for future studies.

## 1. Introduction

Hepatitis C virus (HCV) is a human pathogen, which accounts for approximately 3–4 million new cases of viral hepatitis each year [1]. Scarce data estimate 2–3% of the population worldwide to be chronically infected with HCV. In contrast to hepatitis A, B and E infected patients, chronic hepatitis C patients develop chronic disease (CHC) in most cases (> 80%) [2]. Since the discovery of HCV over 20 years ago, the viral life cycle has been largely elucidated (summarized in [3] and other reviews). HCV is a plus-strand RNA virus, whose genome encodes a single polyprotein, processing of which gives ten mature structural and nonstructural proteins: core, E1, E2, p7, NS2, NS3, NS4A, NS4B, NS5A, and NS5B. The nonstructural proteins comprise an HCV replicase localized to the outer membrane of endoplasmic reticulum (ER), whereas core (nucleocapsid) and glycoproteins E1 and E2 form virus particles. NS5B is the RNA-dependent RNA polymerase whereas NS3 and NS2 proteins exhibit protease activity and are involved in processing of the HCV polypeptide. In addition, NS3 also possesses RNA helicase/NTPase activities that are crucial for replication of the HCV genome. Notwithstanding, all HCV proteins play numerous regulatory functions during HCV replication and formation of infectious particles and/or altering signaling pathways of the host cells.

CHC predisposes to hepatic and extrahepatic diseases including fibrosis, cirrhosis, hepatocellular carcinoma (HCC) and non-Hodgkin lymphoma [2,4,5,6]. In addition, chronic HCV carriers often have various kinds of metabolic dysregulations such as iron overload, insulin resistance and steatosis [7,8,9]. Studies investigating the underlying mechanisms suggest that oxidative stress plays a central role in all these pathologies. These data will be summarized in the current review below.

Oxidative stress is an event of enhanced formation of so called reactive oxygen species (ROS) in the cell [10]. ROS (reactive oxygen species) is a general term indicating a wide set of molecules and radicals including hydrogen peroxide (H_2_O_2_), superoxide anion (O_2_^•-^) and hydroxyl radical (HO^•^). ROS are synthesized in cells by a variety of enzymes in mitochondria, ER, peroxisomes and other cell compartments [10,11].

Eukaryotic cells possess a special system of defense against oxidative stress [12,13]. It is comprised of low molecular weight compounds (glutathione and other antioxidants) and “phase II defense enzymes” capable of scavenging ROS. It is noteworthy that biosynthesis of both phase II enzymes and of the enzymes involved in antioxidant metabolism is regulated by a Nrf2 transcription factor, which recognizes a common conservative sequence in their promoters referred to as ARE (Antioxidant Response Element) [12,13]. The balance between activities of ROS-generating enzymes versus the antioxidant defense Nrf2/ARE pathway determines cellular ROS levels. ROS induce cellular stress either via their direct interaction with various biological molecules including nucleic acids, proteins, and lipids or via activation of classical signaling cascades that regulate stress responses including protein kinases, cytokines, and transcription factors [14,15], which in turn stimulate inflammatory responses. Therefore, understanding the molecular mechanisms of the interplay between HCV and ROS may lead to better comprehension of HCV-induced pathogenicity.

This review aims to summarize current data on how HCV modulates and controls formation and eradication of ROS and the potential roles of HCV induced ROS in the emergence and development of the various pathologies associated with CHC. Discussion of antioxidants as potential antivirals or adjuvants for therapy of CHC is deliberately omitted, as these data are discussed in a recent review by J. Choi [16].

## 2. Oxidative Stress in Patients with Chronic Hepatitis C

Occurrence of oxidative stress during chronic hepatitis C was detected as early as in the middle of 1990s. So far ROS induction by HCV has been put into evidence by various different approaches, including measurement of i) ROS, ii) antioxidants, iii) expression levels and activities of antioxidant defense enzymes, and iv) products of interaction of ROS with biological molecules. Detection of these compounds has been performed in liver biopsies of CHC patients, suggesting a direct influence of HCV, as well as in blood samples or blood cells of CHC patients with special emphasis given to potential correlation between concentration of these compounds/enzymes and course of the disease in the liver. 

Direct measurements in liver tissue from CHC patients revealed an increase of ROS concentrations by two to five orders of magnitude [17,18]. A significant increase was also described in lymphocytes of patients with chronic and occult HCV infection [19]. These data are backed up by a number of papers reporting elevated level of total pro-oxidant activity [20] or of so called “clastogenic score”—ability to modify DNA [21,22] in plasma of the patients.

Glutathione represents one of the major antioxidants, synthesized in all types of eukaryotic cells and especially concentrated in liver [23]. A large proportion of CHC patients has been shown to exhibit reduced levels of glutathione and other antioxidants as well as reduced total antioxidant activity in blood and liver biopsies [20,24,25,26,27,28,29,30,31,32] and others]. In addition to the reduction in the total glutathione level, HCV-induced oxidative stress is accompanied by an increase in the ratio between oxidized (GSSG) and reduced (GSH) forms [31], and enhanced glutathione turnover, at least in peripheral blood mononuclear cells (PBMC) [33].

CHC patients also display decreased levels of antioxidant defense enzymes such as manganese or Cu/Zn superoxide dismutase (SOD), glutathione reductase, and glutathione peroxidase are also often found in PBMCs of CHC patients [19,34,35], although an increase was also reported [36]. Interestingly, expression of the same enzymes is not altered in liver of the same patients, suggesting that the alterations in PBMCs could be a secondary event [36]. CHC patients also display enhanced expression of other defense enzymes including thioredoxin (Trx) [37,38] or heme oxygenase (HO-1) [32].

Lipid peroxidation and advanced oxidation protein products as well as 8-hydroxydeoxyguanosine (8-OHdG) are ROS-modified biological molecules, and were found at significantly higher levels in PBMCs in HCV patients compared to healthy controls ([19,28,29,32,33,34,39,40] and others). Significant increase of 8-OHdG was detected *in situ* in liver samples from CHC patients [41].

## 3. Sources of Reactive Oxygen Species in HCV-Infected Cell and their Regulation by HCV

Various groups aimed to reveal sources of ROS in cells infected with HCV or expressing the individual viral proteins and to unveil the underlying molecular mechanisms. To date, HCV has been shown to activate several different pathways that lead to ROS production, both in hepatocytes and blood cells, which reside in liver.

**Figure 1:**
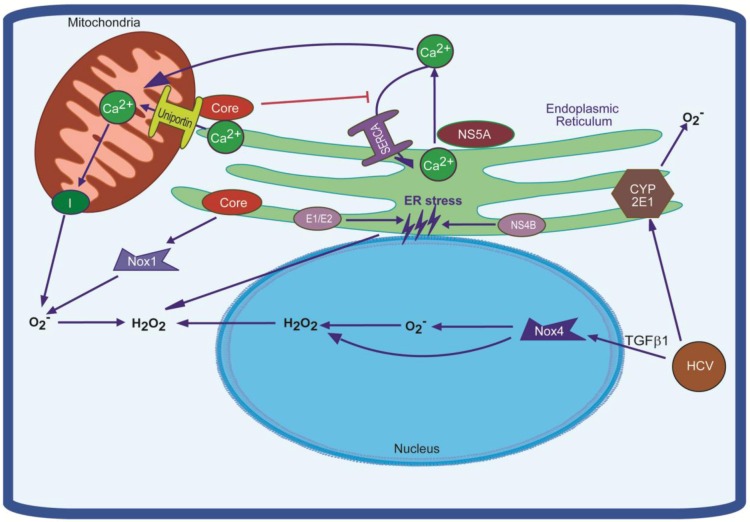
Schematic representation of mechanisms of oxidative stress induction in the HCV-infected cells. They include alteration of functioning of the respiratory chain complex I in response to accumulation of calcium ions in mitochondria. This accumulation is achieved via activation of mitochondrial Ca^2+^ uniporter and enhanced passive leakage of the ions from the ER and suppressed SERCA pump, responsible for Ca^2+^ import into the ER. In addition, Hepatitis C virus (HCV) proteins induces NADPH oxidases (Nox) 1 and 4 which contribute to production of H_2_O_2_ and O_2_^-^. Finally, the ROS (reactive oxygen species) can be generated through ER cytochrome P450 2E1 and induced ER stress. See text for further details.

Most researchers were focused on revealing ROS sources inside hepatocytes. Induction of oxidative stress in these cells has been assigned to almost all HCV proteins: core [42,43,44,45,46,47], E1 [42], E2 [42,48], NS3/4A [43], NS4B [42,49], NS5A [42,43,45,50]. Worth noting is that the HCV core is the strongest regulator [42,43], while NS5A induces early boosts of ROS and reactive nitrosative species (RNS) [45]. To date, two different concepts were approved in the field, which assign ROS production either to NADPH oxidases (Nox) or mitochondria (Figure 1). It has been observed that HCV replication [51,52] or expression of its core protein [44,46,47] lead to mitochondrial dysregulation, often resulting in apoptosis. These mitochondrial alterations are accompanied by massive ROS production due to inhibition of electron transport complex I activity [46,47,51]. Interestingly, this property can be attributed mainly to core protein, since the effect is much more pronounced in the context of the full-length compared to the subgenomic replicon [52]. Mitochondrial dysfunctions are also thought to result from core-induced increase of prohibitin expression, a mitochondrial chaperone which can interact with and regulate expression of mitochondrial respiratory complex IV [53] and possibly electron transport complex I [54]. Importantly, the effects of HCV on mitochondria are not restricted to hepatocytes. Similar effects were also observed in other cell types including lymphoma cells (Raji), expressing HCV core [55], and even in lymphocytes of patients with chronic or occult hepatitis C [19].

Induction of ROS production by HCV has also been shown to be activated through calcium redistribution between ER, cytoplasm and mitochondria (Figure 1). It was shown that chelators of intracellular calcium prevent induction of oxidative stress in cells expressing either the HCV polyprotein [56] or NS4B [49], or core proteins [57]. In core- and NS5A-expressing cells, respectively, two different molecular mechanisms that explain the increase of mitochondrial calcium concentrations have been proposed. HCV core protein has been shown to increase mitochondrial Ca^2+^ uniporter activity [57]. Furthermore, NS5A and core protein have both been shown to deplete ER Ca^2+ ^stores leading to an increase of cytoplasmic Ca^2+^ concentration via induction of a passive leak of calcium ions and inhibition of SERCA, respectively [58,59,60,61]. The latter was shown in various cell lines including Huh7 hepatocytes, Chang liver cells, T-lymphocytes (Jurkat), and HEK293 cells. Finally, calcium redistribution may also be indirectly modified in the context of HCV infection by the presence of ROS, since redistribution can be directly induced by H_2_O_2_ [62] and be suppressed by antioxidants [58].

It could be speculated that localization of HCV core protein is a key factor in causing calcium perturbations, mitochondrial dysfunctioning and ROS production. In line with this hypothesis, the full-length core (1—191 aa) and its mature form (1—173 aa) are known to localize and thus interact directly with the outer mitochondrial membrane [46,63,64], mitochondria-associated membranes (MAM) [65], lipid droplets [66] as well as the ER outer membrane [64]. Mitochondrial localization of the HCV core could be determined by its interaction with the mitochondrial matrix protein HSP60 [67].

A family of NADPH oxidases represents an additional source of ROS in HCV-infected cells. This family is comprised of seven transmembrane enzymes (Nox1-5, DUOX1,2), involved in electron transport through the membranes and thus producing superoxide anion or H_2_O_2_ (Nox4) [68,69]. Interestingly, several NADPH oxidases are activated by calcium signaling [68]. Initially it was shown that human monocytes when incubated with HCV NS3 protein, activate Nox and produce superoxide radicals and other ROS (Figure 1) [70,71]. This process was mediated by calcium ions and p47phox protein [70], which is the organizer subunit of NADPH oxidases 1, 2, and 3 [68]. Later two independent groups revealed that in HCV-infected hepatocytes Nox1 and 4 may act as a primary source of superoxide anion and contribute to production of hydrogen peroxide [72,73]. This was shown both in Huh7 cells transfected with virus-producing JFH1 HCV RNA, and in liver biopsies from CHC patients [73]. Again, HCV core was shown to be the main regulator of Nox4 expression [72]. It is worth noting that activation of Nox4 expression is achieved via TGFβ1 [72,73]. A special emphasis to Nox in induction of oxidative stress in HCV-infected cells is given due to their ability to produce ROS in the nucleus [74,75]. Nuclear ROS in turn can lead to the formation of DNA damage. Indeed, HCV-infected hepatocytes, exhibit increased expression of Nox4 and elevated superoxide levels in the nucleus [73].

ROS production in HCV-infected cells might also arise from other sources than mitochondria or NADPH oxidases. One of such potential sources is the ER-residing cytochrome P450 2E1 (CYP2E1) which is involved in ethanol catabolism [76,77]. It is generally acknowledged that heavy alcohol consumption during CHC leads both to more profound oxidative stress and to aggravated disease progression [78,79]. Indeed, HCV infection was found to enhance expression of CYP2E1 in the livers of at least five CHC-patients with early stage fibrosis [80]. In cell culture systems co-expression of CYP2E1 in HCV core-expressing cells augments ROS production, whereas inhibition of cytochrome enzymatic activity alleviates the stress (Figure 1) [81]. Worth noting is that even in this case ROS were mainly generated not in the ER but in mitochondria [82], which could probably be due to ROS-mediated re-localization of CYP2E1 to this organelle [76].

Increased production of ROS can also arise from ER stress and the unfolded protein response (UPR), which can be induced by a number of chemicals and various viral infections, which alter protein folding or cause ER overload [83]. To tackle ER stress, eukaryotic cells possess a number of defense mechanisms involving expression of various chaperones and other components of the folding machinery, activation of the degradation machinery that targets misfolded polypeptides and suppression of translation [83]. In particular, UPR leads to enhanced expression of protein disulphide isomerases (PDIs) and ER oxidoreductins (Ero1), which are involved in formation of disulphide bond with H_2_O_2_ being a by-product [84]. It was shown that HCV infection [85] or expression of its individual core [60], E1 and E2 [86], and NS4B [49,87] proteins induce ER stress and UPR (Figure 1). Indeed, we observed that HCV E1, E2, and NS4B caused increased ROS production and concomitant activation of antioxidant defense Nrf2/ARE pathway (see below) in a manner similar to tunicamycin, a chemical ER stress inducer [42]. Along with ROS induced by accumulation of unfolded proteins, Ero1 can contribute to oxidative stress by perturbing calcium homeostasis. Recently it has been shown that Ero1α, which is localized at MAM, is involved in regulation of calcium efflux from the ER to mitochondria [88]. Indeed, Benali-Furet *et al.* demonstrated that HCV core causes depletion of ER calcium stores [60]. Therefore, HCV-induced UPR stress may present an additional mechanism(s) contributing to enhanced ROS formation.

## 4. Interplay between HCV and the Antioxidant Defense Nrf2/ARE Pathway

Although oxidative stress during HCV infection was described as early as in 1990s, there have only been few attempts to explore the status of the antioxidant defense Nrf2/ARE pathway. A direct analysis of the status of the Nrf2 transcription factor and of the expression of ARE-dependent genes was first performed by the G. Waris group in 2010 [89]. The study showed that HCVcc infected Huh7.5 cells displayed a sustained activation of Nrf2/ARE pathway from day 2 post-infection (pi) onwards, at a time when core expression had become detectable, until at least 6 days pi. In these cells enhanced *nrf2* gene transcription as well as increased MAP kinase mediated phosphorylation of Nrf2 were observed. An independent study of our group in Huh7 cells demonstrated that activation of the Nrf2/ARE pathway is mediated by five viral proteins, *i.e.*, core, E1, E2, NS4B, and NS5A, the core protein being the most potent regulator [42]. Expression of any of these proteins individually also led to a substantial induction of antioxidant defense, observed as early as 18–20 h posttransfection and lasting at least an additional 20 h. However, we assigned Nrf2 activation to protein kinase C (PKC), casein kinase 2 (CK2), and phosphoinositol-3-kinase (PI3K) [42]. Interestingly, HCV core and NS5A induced Nrf2/ARE pathway activation both by ROS-dependent and -independent mechanisms.

A controversial result was observed by Carvajal-Yepes *et al.*, who demonstrated a suppression of the Nrf2/ARE pathway in the HCVcc system [90]. In this study, the suppression resulted from HCV core and NS3 protein mediated retention of sMAF, a partner of Nrf2, outside the nucleus. These results were obtained in the same cellular background and using the same infection conditions as those used by the group of G. Waris [89], thus the reasons for this discrepancy remain unknown.

Contradictory results were also obtained in a series of papers that regard the influence of the HCV core on HO-1 expression. This Nrf2-dependent enzyme contributes to protection of cells against oxidative stress by converting free heme into Fe^2+^, carbon monoxide, and biliverdin [91]. In Huh7 cells expressing a polypeptide encoding core, E1, E2, p7 and the N-terminus of NS3, elevated HO-1 level were observed, accompanied by suppressed expression of Bach1 protein [92,93], an Nrf2 antagonist in *ho-1* gene transcription [94]. These data were backed up by findings that showed an increase of HO-1 expression in liver biopsies of CHC patients [32]. On the contrary, the group of W. Schmidt reported a marked down-regulation of HO-1 expression, both in Huh7 cells expressing core protein, and in HCV-infected liver biopsies [95,96].

Alternative approaches including transcriptome and proteome analyses did not allow to unambiguously establish the status of the antioxidant defense in HCV infection either. On one hand, an enhanced expression of antioxidant defense genes including microsomal glutathione S-transferase 3 (*mgst3*) and metallothionein 1F was described for the HH4 human hepatocytes transiently expressing HCV polypeptide of 1a genotype [97]. This falls in line with the observed increase in intracellular glutathione content. On the other hand, the same paper reported a suppressed transcription of *sat-1* gene, another Nrf2-dependent gene [98,99], which encoded spermidine/spermine-N^1^-acetyltransferase (SSAT) [97], one of the key enzymes of the metabolism of biogenic polyamines [98,100]. The last observation fall in line with data of S. Blakham *et al.*, which demonstrated a marked suppression of transcript levels of a series of Nrf2-dependent genes including NAD(P)H:quinone oxidoreductase-1 (*nqo1*), catalase (*cat*), epoxide hydrolase 1 (*ephx1*) and of genes driving glutathione metabolism [101]. These data were partially corroborated by the findings of Walters *et al.*, who observed a suppression of antioxidant defense gene expression up to 72 h postinfection [102]. However, they revealed that at later time points (120 h postinfection) transcription of most of these genes is restored to the levels found in uninfected cells or even higher.

It is worth noting that Blackam *et al.*, also revealed a down-regulation of transcription of metallothioneines in HCV-infected cells [101]. Interestingly, transcription of their genes is exclusively driven by Nrf1 [103], which is known to contribute to expression of ARE-dependent genes [104]. Therefore, it is tempting to speculate that HCV might also prevent activation of this ER-localized factor Nrf1, which plays crucial role in protection against oxidative stress [105].

Proteomic analysis of biopsy tissues from CHC patients infected with virus of different genotypes and displaying various grades of fibrosis also did not allow drawing unequivocal conclusion [106]. It was shown that in the liver of these patients expression of antioxidant enzymes varied significantly with no apparent regularity between groups of patients. Hence, additional studies are required to study to role of the antioxidant defense system in CHC disease progression, with a particular attention to be paid to the roles of the HCV genotypes as well as correlations with virological and pathological data.

## 5. Effect of Oxidative Stress on HCV Propagation: Facts and Assumptions

HCV-induced oxidative stress not only contributes to various virus-associated disorders but also affects HCV propagation in the organism and directly modulates several steps of the viral life cycle. However, these data are relatively scarce. Current knowledge suggests that ROS inhibit virus replication without affecting stability of its genome RNA [62,107]. However, ROS can induce viral genome heterogeneity, which facilitates viral escape during treatment [108] and probably escape from the immune system [109]. It is tempting to speculate that increased incidence of hepatitis C chronicity in individuals with null-genotype of glutathione-S-transferases (*gstt1* and *gstm1*) [110] is indeed due to impaired metabolism of glutathione and enhanced oxidative stress. An additional viral escape from immune defense is achieved through ROS-induced suppression of the JAK/STAT pathway leading to resistance to interferon α/β [111].

HCV genome translation is another step in the viral replication cycle which has been shown to be affected by oxidative stress. It was reported that hydrogen peroxide may have no effect on IRES-mediated translation of the HCV genome [107], or can activate this process in sub-lethal concentrations [112,113]. A detailed analysis revealed that the up-regulation was achieved with PKR-like ER-localized eIF2α kinase (PERK) [112], a key player in the UPR [12,114]. As increased ROS production can induce ER stress [115], it could be assumed that the effect of oxidative stress on HCV genome translation is mediated via PERK-mediated inhibition of cap-dependent translation thus favoring initiation of IRES-mediated translation. A suppression of viral genome replication accompanied by enhanced translation could partially underlie the fact that in infected cells there are only few dozens of copies of HCV genome RNA with a huge overproduction of virus proteins [116].

So far the effects of ROS on the HCV life cycle seem to be limited mainly to replication and translation. In contrast, the possible influence of oxidative stress on viral entry and particle assembly and release remain obscure. HCV cell entry is considered a multistep process [117]. Cell attachment is thought to be due to interaction of either HCV-associated lipoproteins with low density lipoprotein receptor (LDLR) and/or interaction of the HCV glycoprotein complexes E1E2 with the cellular attachment receptors CD81 and human scavenger receptor SR-BI. Besides CD81 and SR-BI, the tight-junction factors claudin 1 and occludin have been shown to be required for entry, but in the case of claudin 1 this requirement occurs post-attachment. Upon attachment, the virus is internalized by clathrin-mediated endocytosis which requires the actin and tubulin networks. Many steps of the viral cell entry process may be blocked by oxidative stress. ROS may increase production of oxidized low density lipoproteins, which serve as inhibitors of SR-BI-mediated lipoprotein uptake [118]. Furthermore are claudin 1 and occludin expression sensitive to ROS [119,120]. Finally, oxidative stress products like 4-hydroxynonenal (4-HNE) or 4-hydroxy-2-hexenal (HHE), which are often observed in serum of CHC patients, inhibit tubulin polymerization [121]. Since microtubule remodeling is required for HCV particle internalization [122], alteration of this process with oxidative stress products might block early steps of virus life cycle. It could be also speculated that 4-HNE and HHE might also suppress virus particle assembly and release, since there are recent finding of core protein trafficking along microtubules at later stages of virus life cycle [123]. Thus, investigation of effect of oxidative stress on various stages of virus life cycle presents a challenging task.

## 6. Oxidative Stress and HCV-Associated Diseases

HCV infection is associated with various hepatic and extrahepatic disorders, and oxidative stress contributes to many of them. To date it has been accepted that HCV-induced oxidative stress leads to liver injury. For example, ROS levels, measured in liver using Electron Paramagnetic Resonance Imaging, correlate with histological disease activity although not with levels of serum transaminases [17]. Values of alanine aminotransferase (ALT) and aspartate aminotransferase (AST) positively correlate with various serum markers of oxidative stress [26] and negatively with concentration of vitamin C in plasma [124]. Moreover, an ALT increase in patients who previously had persistently normal levels of transaminases, was preceded by a burst in oxidative stress markers [26]. The level of oxidative DNA modification was also associated with presence and extent of liver damage [39] and with necroinflammatory activity [22,41].

Oxidative stress may play role in HCV pathogenesis during both acute and chronic stages of inflammation. HCV infection leads to activation of immune system and of macrophages in particular [125], is known to result in ROS production by these blood cells [126]. This could be achieved by uptake of HCV components (*i.e.*, dsRNA) by the liver-residing macrophages—Kupffer cells [127]. Activation of Kupffer cells was proposed to contribute to killing hepatocytes via several mechanisms [128]. They include not only enhanced local production of various cytokines including TNF-α [129] but also ROS [130]. However, the latter was shown for other diseases and still has to be verified in case of HCV infection. A recent study of P.A. Knolle and G. Gerken showing that several HCV proteins do induce H_2_O_2_ production by Kupffer cells presents a first evidence confirming this mechanism [130]. In addition, there could be other ways of interplay between HCV-induced inflammation and oxidative stress, and they will probably be one of the key targets for future studies.

### 6.1. Hepatocellular Carcinoma

Oxidative stress is considered as one of the mechanisms, by which HCV promotes proliferation of hepatocytes and triggers HCC. Analysis of oxidative status of CHC patients with and without liver cancer revealed that high Trx and Mn-Sod levels in serum or liver can be used as prognostic markers for detection of HCC [131,132,133]. In addition, 8-OHdG levels could probably be used as markers for HCC recurrence in patients after liver transplantation [134]. Interestingly, increased risk on HCC is associated with a polymorphism in the manganese sod gene, which encodes an antioxidant defense enzyme, at least in Moroccan patients [135]. A direct evidence of cancerogenic potential of HCV-induced oxidative stress was obtained from HCV core-transgenic mice. These animals displayed elevated levels of oxidative stress markers and developed HCC alterations in the absence of inflammation [136,137].

**Figure 2:**
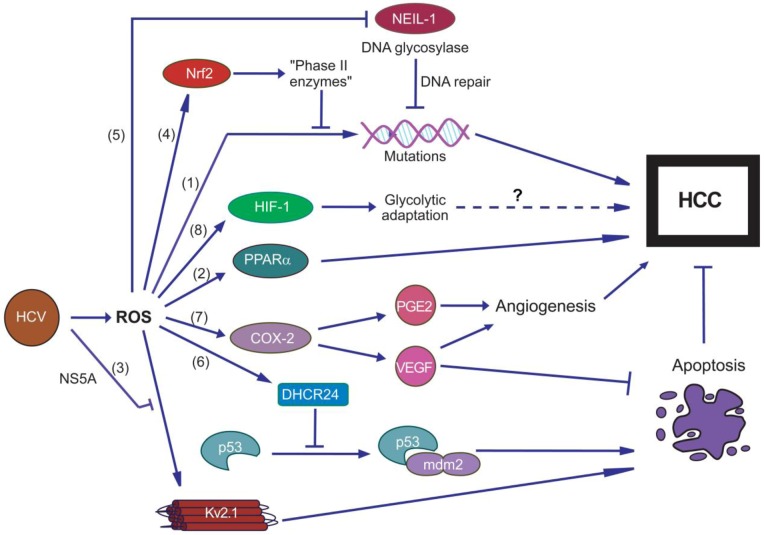
Diagram representing possible mechanisms linking HCV-induced oxidative stress with hepatocarcinogenesis. They include accumulation of DNA damage in response to ROS augmented by suppressed reparation processes (down-regulation of NEIL-1 DNA glycosylase) with concomitant activation of antiapoptotic Nrf2/ARE antioxidant defense pathway. HCV-induced oxidative stress also favors tumor development by enhancing HIF-1—driven glucose uptake and glycolytic adaptation, regulated by hypoxia-inducible factor 1 (HIF-1) as well as by promoting angiogenesis through activation of cyclooxigenase 2 (COX-2) which caused enhanced biosynthesis of prostaglandin E2 (PGE2) and vascular endothelial growth factor (VEGF). In addition, ROS-mediated activation of peroxisome proliferator-activated receptor α (PPARα) as well as blockage by NS5A of proapoptotic potassium ion channel Kv2.1 may also contribute to carcinogenesis.

Currently several mechanisms have been proposed which may contribute to switching cell fate from ROS-induced apoptosis towards tumor occurrence in these animals. First, oxidative stress leads to DNA damage (including 8-OHdG) and leads to accumulation of mutations (Figure 2) [138]. Second, formation of HCC was shown to depend on peroxisome proliferator-activated receptor alpha (PPARα) transcription factor [136], which has been implicated in occurrence of non-virus induced liver cancer [139]. Third, viral NS5A protein acts as an inhibitor of the Kv2.1 potassium channel [140]. In HCV-uninfected cells this channel is involved in induction of apoptosis in response to chemically-induced oxidative stress by amplification of an outward K(+) currant [141]. Therefore, its inhibition by NS5A prevents apoptosis and promotes cell proliferation [140]. Fourth, HCV core-expressing cells have a increased ability to neutralize ROS and are less sensitive to H_2_O_2 _or peroxynitrite probably due to activation of the Nrf2/ARE pathway [42,89,142]. It could be also speculated that additional protection against apoptosis may be attributed to ROS-independent mechanisms of Nrf2/ARE pathway activation, which are achieved through PI3K and CK2 [42]. At least, our unpublished data show that inhibition of these kinases in HCV-core expressing cells leads to a more pronounced cell death (unpublished observations). Fifth, HCV was shown to activate expression of DHCR24, a protein which blocks acetylation of p53 and its interaction with MDM2 and thus inhibits apoptosis [143]. Sixth, HCV-infected cells exhibit altered DNA repair system due to ROS-mediated down-regulation of NEIL1 [43]—a DNA glycosylase involved in excision of oxidized nucleobases from chromatin [144], and are thus prone to the accumulation of DNA damage and liver injuries. Seventh, development of many types of tumors including HCC depends on efficient angiogenesis [145,146]. It was revealed that HCV infection leads to up-regulation of cyclooxygenase 2 (COX-2), increased production of prostaglandin E2 (PGE2), and vascular endothelial growth factor (VEGF) [147,148]—a crucial regulator of angiogenesis and apoptosis prevention [149,150,151]. Interestingly, this was demonstrated using NS5A and core proteins which caused oxidative stress and up-regulation of ROS-sensitive NF-κB transcription factor [50,148]. It is worth noting that PGE2 also contributes to tumor survival by other mechanisms [152], one of which could be activation of antiapoptotic PI3K/AKT signaling pathway [147]. Eighth, tumor growth can be promoted by HCV-induced adaptation of cell metabolism to benefit its survival. In case of HCV it was revealed that mitochondrial dysfunction leads to activation of HIF1α transcription factor, enhanced expression of HIF-1-induced genes involved in glucogenesis, to glycolytic adaptation [153] and increased glucose production [154]. Currently there are no direct evidences of glucose metabolism alterations directly inducing tumor formation, Nevertheless, such alterations are often found in tumor cells, and the compounds targeting glycolytic mechanisms are currently considered as potential anticancer agents [155,156]. Therefore, further attempts are required to unveil the impact of HCV-induced changes in expression of genes involved in glucose metabolisms and tumor formation.

### 6.2. Liver Fibrosis

Liver fibrosis is another hepatic dysregulation induced by oxidative stress in the HCV-infected liver. It was shown that increase in oxidative stress markers such as malonaldehyde and Trx in serum or 8-isoprostane in urine of CHC patients correlates with fibrosis score [22,27,157,158]. In addition, increase in 8-OHdG content in leukocytes is accompanied by increase of the progression speed of liver fibrosis [39]. To date, there is no uniform concept of HCV-induced fibrogenesis. However, extensive studies have revealed several possible mechanisms by which HCV promotes deposition collagen I and other molecules in extracellular matrix (ECM). One of them includes induction of transforming growth factor 1β (TGF1β) in hepatocytes and Kupffer cells in ROS-dependent manner [56,159,160]. This is backed up by several reports which confirm elevated levels of this cytokine in liver and in plasma of CHC carriers with a clear correlation between level of TGF1β and fibrosis score (for example, see [161,162]). A detailed analysis revealed that induction of TGF1β expression is caused by four HCV proteins: core, NS3/4A, NS4B, and NS5A proteins [56] and is achieved via MAP kinase cascades which lead to activation of NF-κB transcription factor [159]. It is worth noting that calcium signaling plays a significant role in both expression of TGF1β and its posttranslational processing, with furin and thrombospondin-1 being the key mediators of the second event [56]. In addition, TGF1β is not the only cytokine involved in fibrogenesis. Recently an independent pathway was discovered. It involved activation of osteopontin, which caused both increase in production of collagen I and suppression of matrix metalloproteinase 13, which is responsible for alleviating scarring [163]. Additional analysis revealed that this pathway was induced via PI3K/AKT/NF-κB pathway [163].

The second concept accounts stimulation of fibrogenesis by uptake by hepatic stellate cells (HSC) of apoptotic bodies derived from HCV-infected hepatocytes [164], by interaction of HSC with the HCV core [165] secreted by the infected hepatocytes, or by E2 protein, expressed in HSC [48]. All this leads to a marked increase in production of TGF1β, connective tissue growth factor (CTGF), collagen I and other profibrotic proteins. In addition, HCV core- and E2-induced events involve induction of oxidative stress, which leads to suppression of matrix metalloproteinase 1 (MMP-1) and other molecules responsible for degradation of the extracellular matrix and reversal of HSC activation [165].

The third mechanism of fibrogenesis is associated with enhanced production of fibromodulin, a proteoglycan by various types of cells including hepatocytes and HSCs [166]. Fibromodulin was shown to promote proliferation, migration, and invasion of HSCs in response to oxidative stress, and CYP2E1-produced ROS in particular. Interestingly, migration of myofibroblast-like cells originating from HSC (HSC/MFs) or from mesenchymal stem cells MSCs was earlier shown for the case of treatment of HSC E2 glycoprotein [167].

### 6.3. Insulin Resistance and Steatosis

HCV infection is also associated with insulin resistance and steatosis. Indeed, consistent correlations between serum Trx and homeostasis model assessment–insulin resistance (HOMA–IR) index have been described [157]. While data on a potential correlation between oxidative stress markers and steatosis, at least in patients with non-3 HCV genotypes [168,169] remain non-conclusive, a clear interrelation between oxidative stress and insulin resistance is highlighted [168]. Currently it is thought that HCV induces steatosis not only via oxidative stress but also by alternative mechanisms, provoked by the same viral proteins. An excellent study shows the effects of mutations in HCV core on both oxidative stress and steatosis [170]. The authors found that presence of glutamine at residue 70 promotes steatosis but not ROS production, whereas methionine at position 91 affects stress levels but not occurrence of steatosis. Search for the mechanisms by which HCV induces insulin resistance revealed increased expression of peroxisome proliferator-activated receptor-gamma co-activator 1α (PGC-1α) both in HCV-infected cells and liver biopsies from CHC patients [154]. This protein is a transcriptional co-activator of a set of genes involved in initiation of gluconeogenesis [171,172] and is implicated in the induction of insulin resistance in response to oxidative stress [173]. ROS-mediated elevation of its transcript levels was accompanied by up-regulation of glucose-6 phosphatase (G6Pase) and increased glucose production [154]. Other ROS-induced factors that may contribute to insulin resistance and steatosis are an enhanced uptake of fatty acids, observed in HCV core-transgenic mice ascids [136], or Sterol Regulatory Element Binding Protein-mediated up-regulation of genes involved in cholesterol and lipid synthesis [174]. However, there could still be other yet undiscovered mechanisms implicated in induction of steatosis and insulin resistance by HCV.

## 7. Oxidative Stress and Iron Overload: “The Chicken or the Egg” Dilemma

Currently much attention is being paid to alterations in the iron homeostasis in HCV-infected patients, as these changes can promote oxidative stress and may contribute to the associated pathologies. Iron ions are involved in ROS production through Fenton’s reaction, which consists in conversion of a low active H_2_O_2_ into highly active hydroxyl and peroxide radicals [10,175]. Therefore, increase of iron levels is regarded as potential cause for oxidative stress. Iron ions are stored in several organs and tissues, liver being one of the main depots in human body [176,177,178]. Dietary iron is adsorbed from the duodenum in the form of Fe^2+^ ions by a divalent metal transporter (DMT1). In cells, iron exists mainly as iron-ferritin complexes [177,178]. Ferritin is a multisubunit protein shell, which can store up to 4500 iron atoms [176]. Iron concentrations in hepatic cells are regulated by the transferrin receptor (TfR) and ferroportin, which control influx and efflux of iron-ferritin complexes respectively [177,178]. Another key factor, which orchestrates iron homeostasis, is hepcidin, a 25 aa peptide hormone, produced mainly in hepatocytes [176,178]. Upon increase of its level, hepcidin binds to ferroportin and DMT1, thus targeting them for degradation and consequently blocks iron efflux from the cells and absorption from the duodenum [176,178]. Expression of hepcidin is controlled at the transcriptional level and is activated in response to an increase of iron concentrations [178].

CHC patients often display altered iron homeostasis. App. 40% of patients have elevated levels of iron and ferritin in the serum, and 10% of patients have elevated levels of iron in the liver [7]. Moreover, iron deposition in the liver is enhanced during treatment with ribavirin [179]. Currently this is linked to two factors: patients’ genetics and direct influence of HCV-infection. It was shown that CHC patients with mutations in hemochromatosis (HFE) gene [180,181,182,183] or β-globin [184,185], or in the hepcidin promoter [185] have higher iron levels in the liver, and more severe fibrosis and cirrhosis. Furthermore, HCV replication also affects iron metabolism. CHC patients exhibit enhanced expression TfR1 and -2 in the liver [186,187], which is accompanied by a decrease in serum hepcidin levels [188]. In *in vitro* replication systems it was also shown that HCV replication leads to suppressed expression of TfR1 and increased expression of ferroportin, thus leading to altered iron uptake and its enhanced leakage from Huh7 cells [189]. Interestingly, alterations of iron homeostasis are affected by HCV genotype: genotype 3 virus leads more frequently to hepatic iron deposits [190]. Dysregulation or iron homeostasis is likely to result from alteration of expression of hepcidin, as suggested by two observations. First, hepcidin serum mRNA levels correlated with iron storage in liver and serum ferritin [191,192]. Second, in cell culture systems a suppression of hepcidin expression was observed, with the core protein acting as a regulator [193]. It is worth noting that this down-regulation was achieved through ROS-mediated activation of histone deacetylase (HDAC) which caused hypoacetylation of histones and reduction of binding of C/EBPalpha and STAT3 transcription factors to hepcidin promoter [193].

The opposite question, *i.e.*, influence of iron on HCV reproduction, is controversial. There are several papers in the field, reporting stimulation [194], suppression [195,196], or no effect [197] of iron on HCV replication, as well as activation of IRES-mediated translation [198,199]. Notably, inhibition of HCV replication was shown both in replicon and HCVcc systems [195,196], with a number of underlying mechanisms proposed. According to one of them, suppression could result from inhibition of enzymatic activity of the viral RNA polymerase NS5B [195]. Alternatively, iron induces HO-1 [93], the product of which, biliverdin, is known to activate interferon responses [197] and to block NS3/4A protease activity [200]. In line with this, an iron donor hemin, displays anti-HCV activity acts synergistically with other anti-HCV agents [201].

Numerous studies in humans and in animal models revealed that the alteration of iron homeostasis is closely associated with various HCV-induced pathologies including liver fibrosis [202,203], steatosis [190,204,205], insulin resistance [206], diabetes mellitus [207], porphyria cutanea tarda [208], and HCC occurrence [205]. In contrast, iron deposition in liver does not correlate with rates of sustained virologic response (SVR) during interferon/ribavirin therapy [209,210], although there is some evidence of a negative correlation between SVR and the level of serum ferritin [209]. At the same time several studies reported the same relationship between alterations of iron metabolism and interferon monotheraly [210,211,212]. Phlebotomy in CHC patients leads to a decrease in hepcidin expression [213] and to a marked reduction of serum transaminases ALT and AST and liver injury [211,214,215]. However, it is considered that reduction of iron overload does not lead to increase in SVR rate [216,217,218,219], although opposite has also been reported [220].

Iron overload and oxidative stress during HCV infection are closely related to each another. There is a strong correlation between serum ferritin levels and lipid peroxydation markers in CHC patients [31]. 8-OHdG content, a DNA oxidation marker, also correlates with hepatic iron storage markers including serum ferritin, hepatic total iron score and hepcidin mRNA levels [221]. In addition, transgenic mice expressing an HCV polyprotein and subjected to iron-rich diet also exhibit signs of oxidative stress in liver (lipid peroxydation, 8oxoG), accompanied by alterations of mitochondrial ultrastructure [205]. Currently there is no consensus on which of them is a trigger of another. On one hand, suppression of hepcidin expression is mediated by ROS [193]. On the other hand, phlebotomy or dietary iron restriction reduces oxidative stress and lipid peroxidation in CHC patients [215,221].

## 8. Future Directions

Since the discovery of HCV, numerous pieces of evidence have been obtained that show that HCV infection leads to strong oxidative stress. This stress has been linked to several HCV-associated disorders including HCC, insulin resistance and steatosis, liver fibrosis and iron overload. Although several molecular interactions between distinct HCV proteins and ROS-generating enzymes have been identified, additional mechanisms by which HCV induces oxidative stress still remain to be discovered. In addition, discrepancies between the data regarding the status of the antioxidant defense system, obtained in cell culture systems and in vivo in CHC patients, require further studies. Perhaps, the conflicting data may be resolved by investigation of dynamic changes in the Nrf2/ARE pathway regulation throughout the course of HCV infection.

Investigation of HCV-induced oxidative stress has been mainly focused on its role in neoplastic transformation of hepatocytes so far. In contrast, the role of HCV-induced oxidative stress and its interplay with metabolism of various classes of compounds remains ill defined. In the coming years attempts to unveil the impact of enhanced ROS production on alterations of the metabolism of carbohydrates, lipids and lipoproteins, amino acids and polyamines, as well as cytokines and the immune system will be of vital importance and a technical challenge in the field, as these insights may help to unravel the role of these events in disease progression towards hepatocarcinogenesis. 
